# New Drugs and Preparations for the Sick

**Published:** 1917-04

**Authors:** 


### New Drugs and Preparations for the Sick.

---

**Vitafer** (Southall Bros. & Barclay) is manufactured from milk with the addition of various glycerophosphates. It is stated to yield the following composition on analysis:—

| | |
|---|---|
| Casein and Lactalbumen .. .. .. .. .. .. | 85.0% |
| Milk Fat .. .. .. .. .. .. .. .. .. | 2.0% |
| Inorganic Salts (mainly calcium phosphate) | 7.5% |
| Glycerophosphates of Sodium .. .. .. | 1.1% |
| ,, Calcium .. .. .. | 2.2% |
| ,, Magnesium .. .. .. | 2.2% |
| Carbohydrates, *e.g.* Sugar, Starch .. .. | absent |
| Purine producing substances .. .. .. .. | absent |
| | 100.0% |

We need not point out that such a food has many uses, or that it fulfils the purpose of food and tonic. It is recommended for administration in milk, cocoa, coffee, tea, porridge, etc., in fact any *non-acid* liquid food, hot or cold, as it breaks down in acid solution.

**Lycresol** (Southall Bros. & Barclay) is stated to be a mixture of cresylic acid and soft soap, containing fifty per cent. of free cresol. It should prove a valuable and efficient disinfectant for sterilizing instruments, as a douche or lotion in suitable dilution, and for general purposes.

**Cystoforium** (Southall Bros. & Barclay).—As a powerful urinary antiseptic and mild diuretic is indicated in infected conditions of the urinary tract. We understand that it is a compound of hexamethy lenetetramine with sodium acetate, with forty per cent. of its weight of the former. It may be prescribed in tablets, one to three thrice daily dissolved in water.

**Hippurates of Ammonium, Lithium and Sodium** (Southall Bros. & Barclay) have been introduced for the treatment of persistently abnormal high blood pressure due to various causes, where hitherto one has administered iodide of potassium or the nitrites. Their use seems less liable to cause depression than the iodides, while more prolonged in action and less active than the nitrite group.

**Aldemint** (Southall Bros. & Barclay).—We have become familiarised with the use of lozenges containing formaldehyde as an antiseptic in various conditions of the mouth and pharynx. The tablets introduced to our notice are pleasant, and will be found of great utility in suitable cases.

